# All-in-One Spinal Cord Stimulation in Lymphoproliferative Diseases

**DOI:** 10.3389/fneur.2020.550554

**Published:** 2020-11-13

**Authors:** Antonello Sica, Beniamino Casale, Caterina Sagnelli, Maria Teresa Di Dato, Pietro Buonavolontà, Anna Maria Salzano, Evangelista Sagnelli, Vincenzo Famiglietti, Elisabetta Saracco, Dario Tammaro, Alfonso Papa

**Affiliations:** ^1^Department of Precision Medicine, University of Campania Luigi Vanvitelli, Naples, Italy; ^2^Department of Pain Therapy Monaldi Hospital—Azienda Ospedaliera di Rilievo Nazionale Ospedali dei Colli, Naples, Italy; ^3^Department of Mental Health and Public Medicine, University of Campania Luigi Vanvitelli, Naples, Italy

**Keywords:** lymphoproliferative diseases, spinal cord stimulation, chronic pain, burst stimulation, DRG stimulation

## Abstract

Even patients with lymphoproliferative diseases may develop a persistent chronic pain not responsive to usual treatments due to changes in antibody production and to some treatments like radiotherapy, chemotherapy, and the administration of monoclonal antibodies, which further impair the immune defense and induce chronic inflammatory phenomena acting as a substrate for a persistent chronic pain. Five patients with indolent lymphoproliferative diseases were treated for severe pain nonresponsive to other pain reliever treatments with SCS applied with an All-in-One Shot (OS) procedure. For all patients, the estimated survival time was of 5 years or more. All patients showed a significant reduction of the intensity of pain: the mean Numerical Rating Scale was 7.4 before treatment and 2.2 after. No patient developed adverse events. Supported by the data of this study, we believe that the habit to deprive patients with an indolent form of lymphoproliferative diseases of the possibility to reduce the intensity of chronic pain by SCS treatment is extremely reductive and frustrating.

## Background and Introduction

The pain is an epiphenomenon of several pathological processes. A constant stimulation of the nociceptive pathways produces changes in the ion channels, receptors, and nerve synapses, which, together with an increased diffusion of neurotransmitters and neuromodulators, facilitates the depolarization of peripheral and central neurons (1–5). Thereby, ectopic discharges are generated that activate and amplify the neighboring cells generating a constant painful sensation. These mechanisms also involve the dorsal root ganglia, where the sensory neurons reside and/or the dorsal horn of the spinal cord, which receives peripheral inputs to modulate pain sensitivity. In addition, the overproduction of cytokines (IL-6, TNF-α, and IL1-β) by macrophages and monocytes amplify the inflammation and, consequently, induces an increase of pain (6–8). Fortunately, both activated B and T lymphocytes exert an inhibitory action on pain by the production of IL-10, the cytokine with the highest inhibitory action on IL-1β, IL-6, and TNF-α secretion (9–12). In addition, in diseases inducing an alteration of the immune system, like in lymphomas, the abovementioned mechanisms may be amplified with a consequent enhancement of pain (13–16).

The subcutaneous spinal cord stimulation (SCS) is among the main innovative techniques to control the otherwise intractable chronic pain. SCS uses low voltage current that reaches the spinal cord through the application of epidural leads that stimulate nerve fibers blocking or weakening the transmission of pain (17–21). The subcutaneous SCS induces a tingling sensation in the patient that overlaps the area where the pain is felt. High-frequency SCS without paraesthesia has been introduced for treatment chronic pain and has shown good efficacy (22). The location where the stimulator needles are introduced varies according to the metamer involved in pain production. The system is considered effective if the patient perceives a reduction of pain of at least 50%. Usually, a test device is first used, which, if valid, is replaced with a definitive system consisting of housing the neurostimulator (pulse generator) in a subcutaneous pocket, abdominal or lumbar. The pulse generator is then connected to the subdural lead by means of a connection cable that is tunneled into the subcutaneous tissue, so that no component of the definitive system remains externally visible at the end of the intervention (23–27). The complications due to the surgical insertion of an epidural lead are usually reversible and include bleeding with hematoma formation in the epidural space, headache due to accidental puncture of the *dura mater* with loss of cephalorachid fluid, infections and paresis due to possible compression of the spinal cord, extravasation of serum at the pulse generator plant site, feeling of numbness or persistent pain localized to the area below the implant or at the electrode implant site, allergic reactions, and attempts of rejection for implanted materials. Over time, a stimulation change may occur due to cellular changes in tissues surrounding the electrodes caused by the displacement of the electrodes, loosening of the electrical connections, defects in the lead, or alterations in the charge of the batteries. Spinal cord stimulation is mainly applied in the failed back surgery syndrome (FBSS), neuropathic pain, peripheral ischemic pain, amputation pain and visceral pain (28, 29), and disorders involving chronic pain lasting for years.

Lymphoproliferative diseases can be classified as aggressive or indolent. The aggressive forms need immediate intensive treatment to improve the expectancy of life. Instead, Indolent lymphoproliferative diseases have a slow growth and are usually treated only in case of systemic symptoms, symptomatic splenomegaly, bulky mass over 7 cm, and progressive leukemic serum effusions. The indolent forms require a 3- to 6-month watch-and-wait period to verify whether the disease may turn aggressive; this strategy is linked to three main reasons: the good long-term prognosis measured in years, the absence of benefits with chemotherapy that however may induce severe side effects, and the possibility of these indolent forms going through multiple reactivations over time requiring different chemotherapy treatment (30, 31). Chronic lymphocytic leukemia/small lymphocytic lymphoma (CLL/SLL), Waldenström macroglobulinemia (WM), follicular lymphoma (FL), and marginal zone lymphoma (MZL) are all examples of indolent lymphoproliferative diseases (32–34). International Prognostic Scores to forecast the survival expectation of each single type of lymphoma have been proposed and validated. The parameters used in these systems are the clinical stage, the number of sites involved, the age of patients, blood count, immunoglobulin heavy chain region variable (IGHV) mutational status, cytogenetic abnormalities, serum β2 microglobulin, lactate dehydrogenase (LDH) serum values, and serum monoclonal protein concentration. The performance status and the presence of comorbidities should also be considered, conditions that may influence the survival in patients with lymphoproliferative diseases. An imbalance in antibody production is commonly observed in these patients. This immunologic damage is aggravated in treated patients using immunosuppressants (chemotherapy, monoclonal antibodies, and radiotherapy) with a stronger reduction in the efficacy of the immune system and with an increased possibility to develop serious infectious diseases. This requires remarkable caution in carrying out SCS in patients with lymphoproliferative diseases. A cause–effect relationship between trial duration and the risk of infection is well-demonstrated (35); in this study, we describe the effect of All-in-One SCS in five patients at elevated infection risk with an indolent lymphoproliferative disease complaining of an intense pain resistant to numerous pain-relieving treatments.

## Materials and Methods

In the last 2 years, five patients with B-cell lymphoproliferative diseases with a life expectancy of more than 5 years complaining for an intense persistent pain resistant to numerous pain-relieving treatments were treated with SCS at the Department of Pain Therapy Monaldi Hospital—AORN Ospedali dei Colli, Naples, Italy. Before implanting the SCS apparatus, individual prognostic scores were examined.

The International Prognostic Score (CLL-IPI) was calculated for two CLL patients, the FLIPI was calculated for two follicular lymphoma (FL) patients, and the International Prognostic Scoring System (ISSWM) was calculated for one patient with WM. The SCS stimulation was considered effective when a pain reduction >50% was obtained. The intensity of pain was assessed according to the McGill Pain Questionnaire (MPQ), recording the Numerical Rating Scale (NRS) of the pain average: minimum pain, maximum pain, and pain during exertion (scale 0–10, where 0 = no pain and 10 = worst pain ever). At the baseline visit, each patient signed an informed consent for the anonymous use in clinical investigation and scientific publication, according to Italian laws on privacy and with the rules of our ethics committees. All patients were evaluated with ECOG Performance Status (PS). Patients, according to the CARE guidelines, underwent physical examination, routine blood chemistry tests, and serum markers of HBV, HCV, and HIV infections (by commercial immunoenzymatic assays) (36–42). To reduce the risk of infection, the SCS apparatus was implanted in a single session (One Shoot technique).

### Statistical Analysis

The Wilcoxon signed-rank test was used to evaluate changes in NRS before and after SCS in each patient, a non-parametric test useful for assessing whether two repeated measurements in a single patient differ significantly (*p* < 0.05). We assessed individual NRS changes in pain before and after SCS with the Wilcoxon signed-rank test using the Reliable Change Index, which indicates whether a change in the NRS score is significantly greater than expected based on the reliability test–retest. In the reliable change, index X1 represents a subject's pre-test score, X2 represents the post-test score in the same subject, and Sdiff is the standard error of the difference between the two test scores. Sdiff can be computed directly from the standard error of measurement (SE) according to the following: RC: X1 – X2/SDIFF, where SDIFF = √2(SE) 2, and SE is computed as follows: SE = s√1 – rxx. We used a 95% confidence interval, meaning that we considered RCI values of 1.96 or higher statistically significantly.

### Brief Report of the Cases

**Figure 1:**
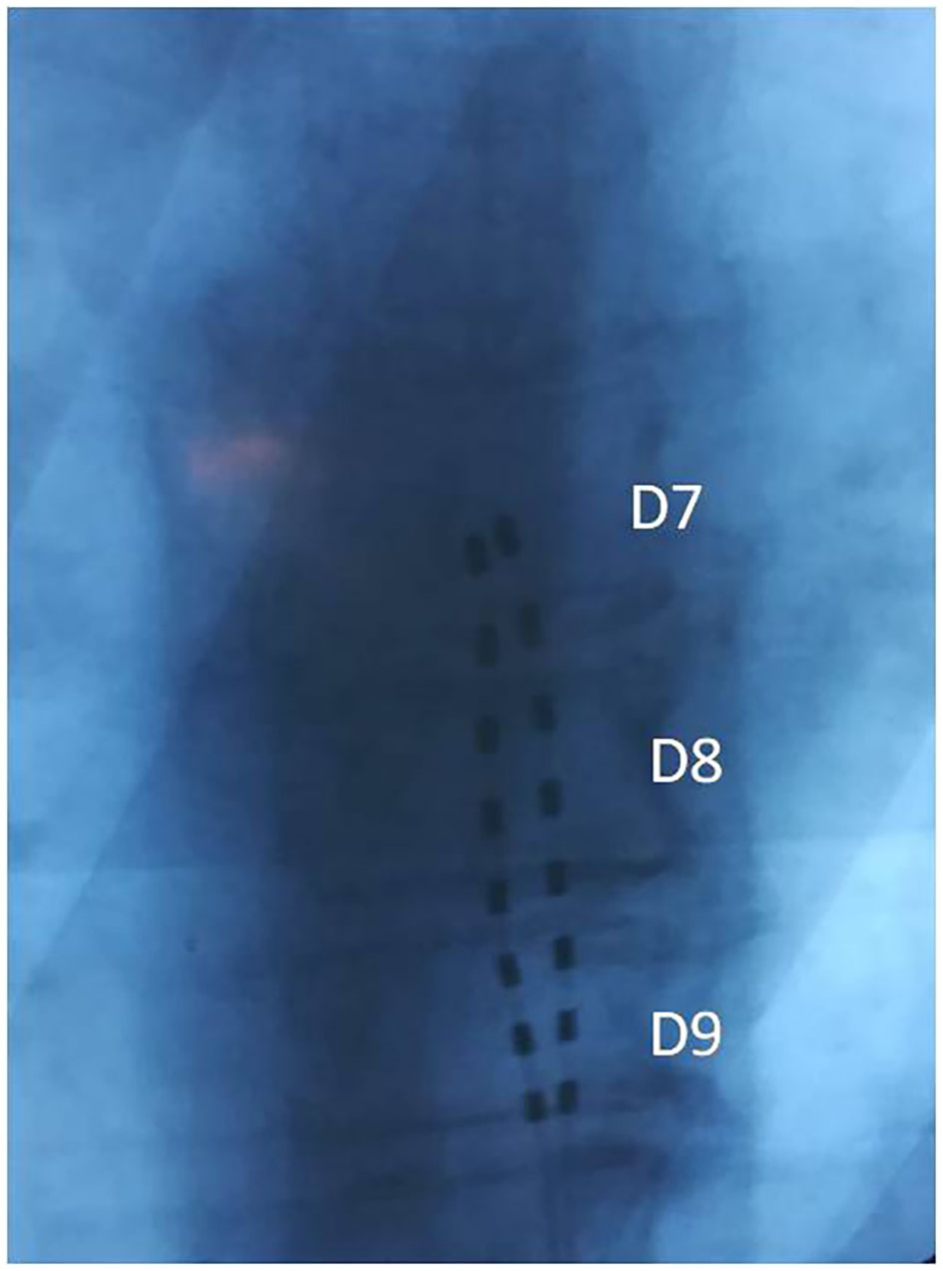
Case 1: Two aligned octopolar leads with tip in T7.

**Case 1:** A 70-year-old Caucasian man was first observed at our clinical center in January 2019 for chronic lymphocytic leukemia stage II according to Rai staging. This patient complained about a left leg pain with a NRS score of seven due to a FBSS consequent to microdiscectomy for lumbar disc herniation performed 1 year before. The only blood test abnormalities were a lymphocytosis (15,000 cells per mmc) and a slight decrease in gamma globulin serum levels. He had an IGHV mutated status, no cytogenetic abnormalities, serum β2 macroglobulin = 2 mg/L, and PS = 2. His CLL-IPI showed an intermediate risk with 79.3% 5-years survival possibility. He was negative for serum markers of HBV, HCV, and HIV infections. In February 2019, he was treated with OS dorsal SCS, implanting two leads with tip in T7; the chosen waveform was BURST DR with the following parameters: frequency 40 Hz, intraburst 500 Hz, pulse length 1,000 ms, Max Amplitude 2 mA (Figure 1). His pain was relieved soon and the NRS score was 2 in March and in August 2019. No adverse event had occurred.

**Figure 2:**
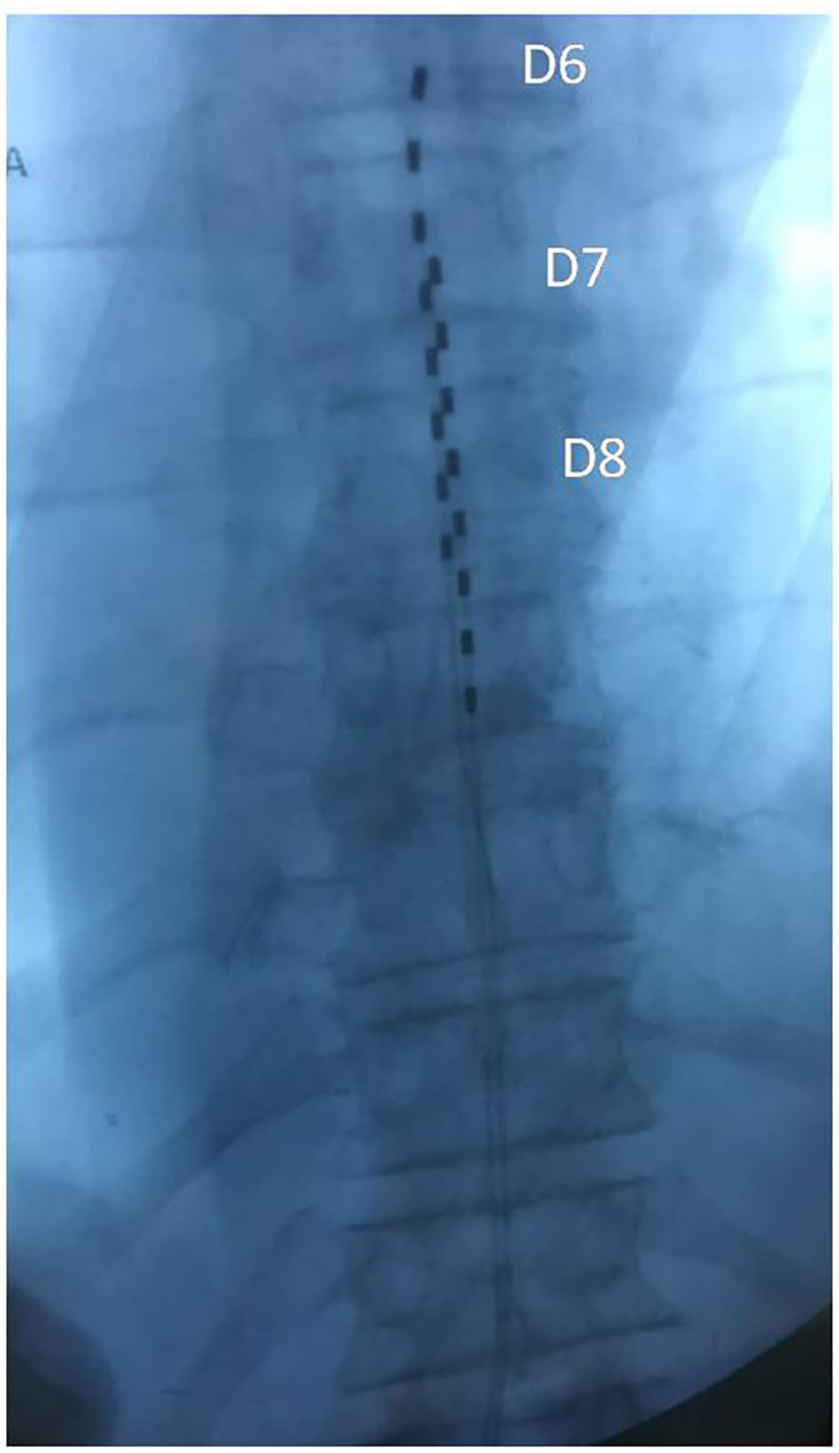
Case 2: Two octopolar leads with a non-aligned tip in T6 (left) and T7 (right).

**Case 2:** A 65-year-old Caucasian woman was observed at our clinical center in March 2019 for a back pain with multiple radicular irradiation not responsive to treatments with oral opioids and multilevel dorsal root ganglion (DRG) pulsed radiofrequency treatment (PRF). She was affected by CLL stage II according to Rai staging. A lymphocytosis (30,000 cells per mmc) and a slight decrease in gamma globulin serum levels were the only abnormalities in the blood tests. She had an IGHV mutated status, no cytogenetic abnormalities, serum β2 microglobulin = 3 mg/L, and PS = 2. Her CLL-IPI showed a low risk with a 93.2% possibility of 5-year survival. He was negative for serum markers of HBV, HCV, and HIV infections. The NRS was 8 in April 2019 when she was treated with an OS dorsal SCS implanting two leads with a non-aligned tip in T6 (left) and T7 (right); the chosen waveform was BURST DR with the following parameters: frequency 40 Hz, intraburst 500 Hz, pulse length 1,000 ms, Max Amplitude 2 mA (Figure 2). Her pain was relieved soon and her NRS score was 2 in May and in November 2019. No adverse event had occurred.

**Figure 3:**
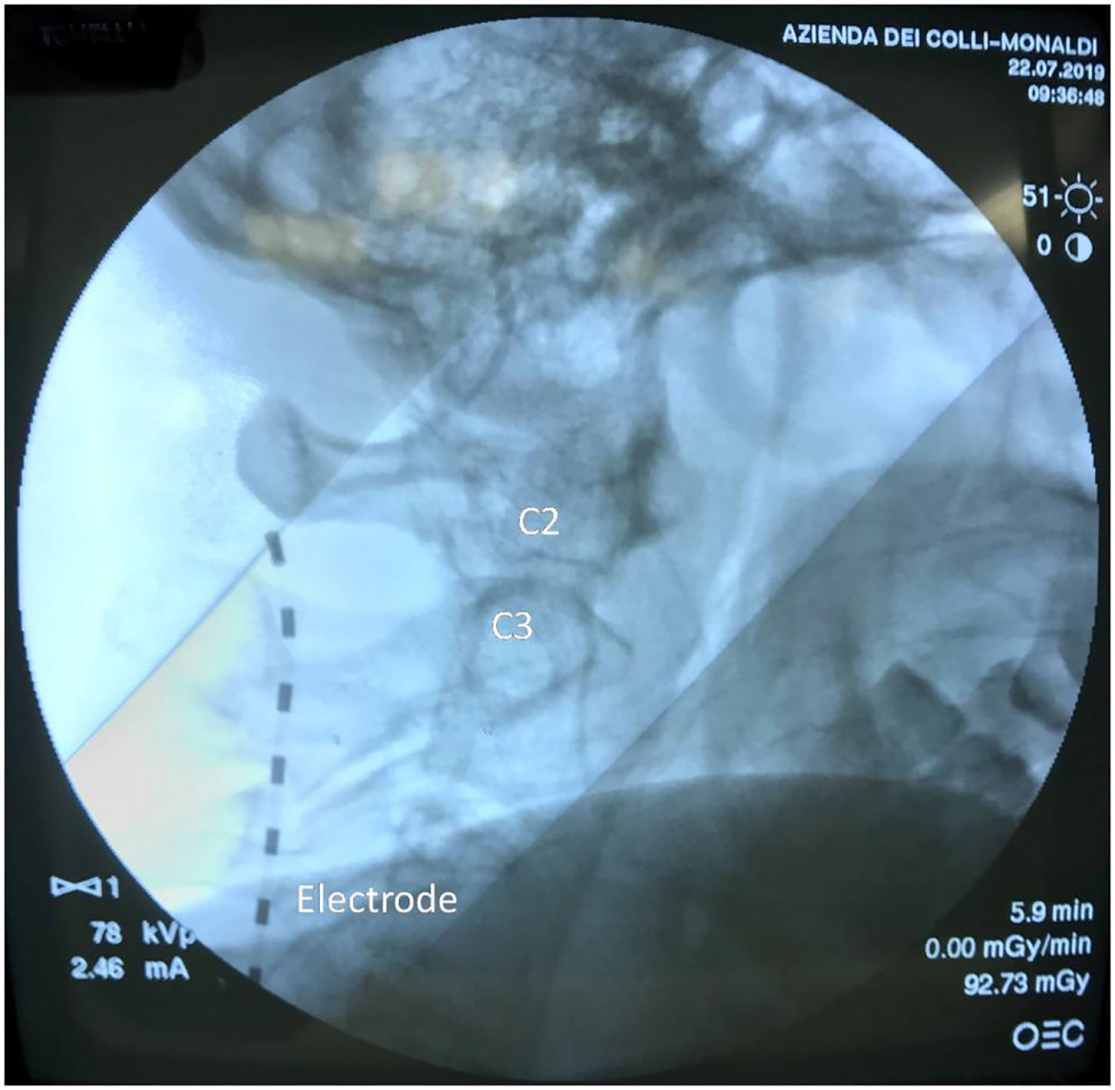
Case 3: Dedicated octopolar high-frequency lead with tip in C2–C3; the sweet spot of the electric field was set in the e1–e2 dipole.

**Case 3:** A 53-year-old Caucasian woman was observed at our clinical center in June 2019 for a severe herpetic trigeminal neuropathy involving both the first and second branch non-responsive to carbamazepine, opiates, and local anesthetic therapy persisting for 2 years. At that time, a severe vesicular eruption was also present and the NRS was 10. She was suffering from myasthenia gravis on treatment with immunosuppressants for 20 years and from WM in a watch-and-wait therapeutic program. Her blood count and blood chemistry tests were normal, except for a broad-based peak in the gamma globulin region. Her serum IgM was 1.8 (g/L) and serum B2-microglobulin = 2.2 (mg/L). Her ISSWM was 0 with a median survival of 142 months. Her PS was 2. All serum markers of HBV, HCV, and HIV infections were negative. She was treated with an OS SCS implanting one lead with tip in C2–C3 in July 2019; the chosen waveform was high frequency at 10 kHz; the sweet spot of the electric field was set in the e1–e2 dipole (Figure 3). Her NRS score decreased to 5 in August 2019 and to 4 in February 2020. No adverse event was observed.

**Case 4:** A 70-year-old Caucasian man was observed at our clinical center in April 2018 for a severe herpetic thoracic neuropathy non-responsive to carbamazepine, opiates, and local anesthetic treatments persisting for 1 year with a NRS score of 6. He was also affected by FL stage II according to Ann Arbor staging with two sites involved. His PS was two. The blood cell count and the other blood chemistry tests were normal, with a mild depression in the gamma globulin region. His FLIPI was 1, suggesting a low-risk category and a 10-years overall survival of about 70%. Serum markers of HBV, HCV, and HIV infections were negative. She was treated with right T8 and T10 DRG stimulation in May 2018; the implant was applied with an OS procedure. No adverse reaction had been observed. His NRS was 1 in June 2018 and 2 in December 2018 and June 2019.

**Case 5:** A 69-year-old Caucasian man was observed at our clinical center in September 2019 for a severe intercostal neuropathy that arose after a diagnostic lung biopsy performed in a video-assisted thoracoscopic surgery (VATS) for pulmonary FL. No other side of the disease was demonstrated by a [^18^f] fluoro-D-deoxyglucose positron emission tomography/computed tomography (FDG-PET/CT) performed in February 2019. At that time, the LDH serum value was 200 IU/L, and all other blood tests were normal. In March 2019, he performed a RT (24Gy) and in May 2019, a FDG-PET/CT showed a complete remission of pulmonary lesion.

**Figure 4:**
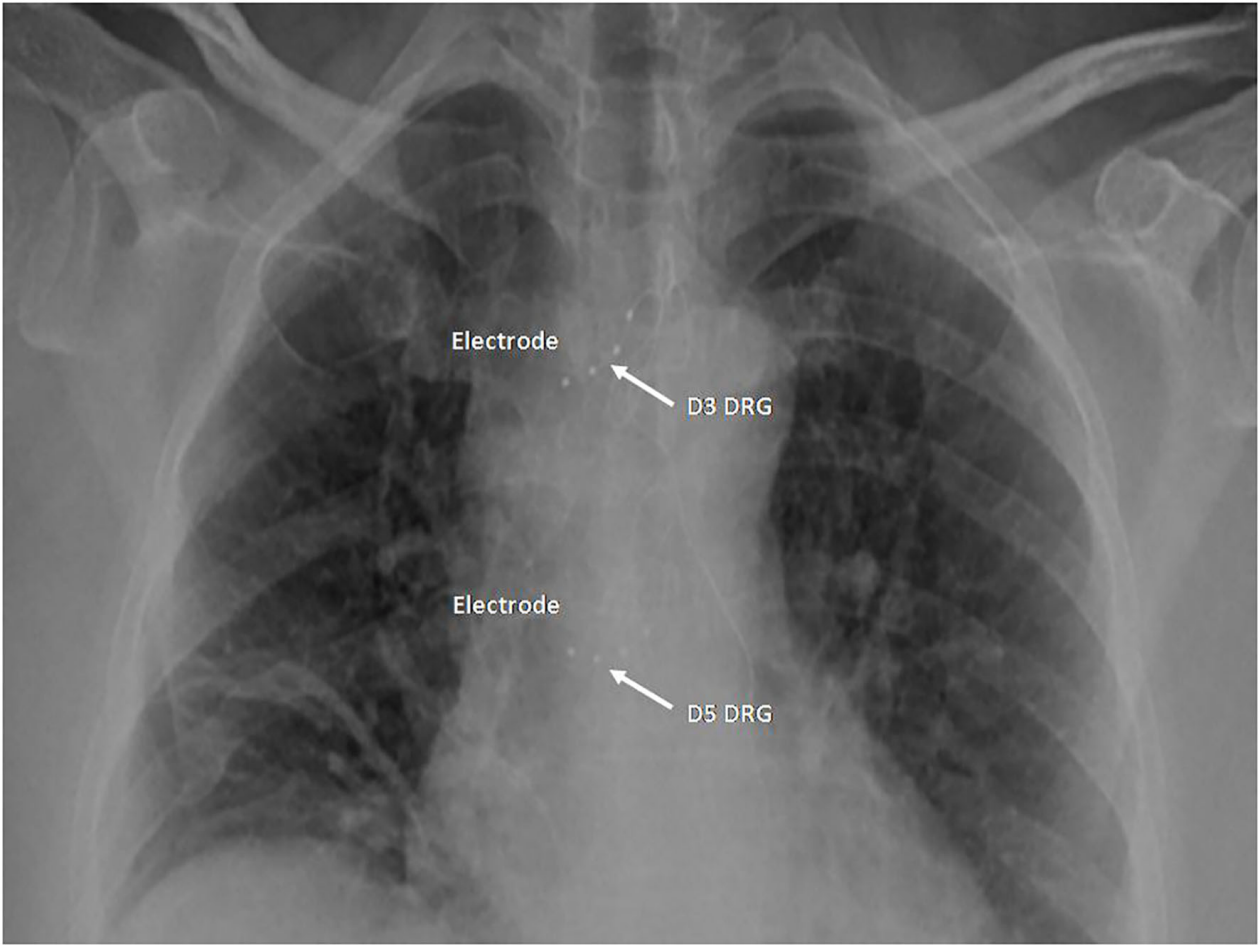
Case 5: Two quadripolar leads in right D3 and D5 foramen.

No pain relief was observed after DRG pulsed radio frequency (PRF) thermal ablation of the transverse process of D4 performed in May 2019. The patient was in stage IE according to Ann Arbor staging, his PS was two, and his FLIPI was one, a value indicating a low-risk category with a 10-year overall survival of about 70%. Serum markers of HBV, HCV, and HIV did not show an ongoing infection, while an anti-HBc positivity was a clue of a past HBV infection. In September 2019, still suffering for an intractable persistent intercostal pain with NRS of 6, he was treated with right D3 and D5 DRG stimulation. The implant was done using an OS procedure with the following parameters: continuous stimulation frequency, 30 Hz; pulse width, 300 ms; max amplitude range, 0.350–0.450 mA (Figure 4). The NRS was 1 after the SCS and was also 1 in March 2020. No adverse event had occurred.

### General Comments on the Five Clinical Cases

Five patients with lymphoproliferative diseases were treated for severe pain nonresponsive to other pain reliever treatments with SCS, two had CLL, one had WM, and two had FL; two were females and three were males; the median age of the patients was 69 years (range 53–70). The PS was two in all cases. The SCSs implanted were lumbar in one patient, cervical in two, and ganglionic in two. For all patients, the estimated survival time was of 5 years or more. The mean NRS before treatment was 7.4, with highest peak at 10 and the lowest at six, whereas the mean NRS after treatment was 2.2, with highest peak at five and lowest at one (Table 1). The Wilcoxon signed-rank test concerning NRS changes before and after HFSCS showed a significant favorable difference between the two time points (*z* = −2.12; *p* = 0.034). The reliable change index for NRS score at the two different time points showed a RCI of 2.07 and an effective size of 3.11. These data confirm that HFSCS has produced a significant reduction of pain in our five patients.

**Table 1:** Some characteristics of five patients with lymphoproliferative disease and chronic pain treated with SCS.

**No. of Patients**	**Disease**	**Sex**	**Age**	**PS**	**Prognostic index**	**Expected survival**	**NPR score before TX**	**NPR score after TX**
1	CLL	M	70	2	CLL-IPI 1	5-years survival: 79.3%	7	2
2	CLL	F	65	2	CLL-IPI 2	5-years survival: 93.2%	8	2
3	WM	F	53	2	ISSWM 0	Median survival: 142 months	10	5
4	FL	M	70	2	FLIPI 1	10-years overall survival: approximately 70%	6	1
5	FL	M	69	2	FLIPI 1	10-years overall survival: approximately 70%	6	1

We conclude that the high-frequency SCS was useful for all patients with lymphoproliferative diseases included in the present study.

No patient had ongoing HIV, HCV, and HBV infections. None of these five patients experienced adverse events or infections signs.

## Discussion and Conclusion

The most common indications for SCS are FBSS, complex regional pain syndrome, brachial plexopathy, diabetic neuropathy, post-herpetic neuralgia not responding to other treatments, and pain associated with vascular pathology or secondary to damage of peripheral nerves. All these indications do not foresee their use in the onco-hematological patients. In these patients, the greater risk of both systemic and local infections could jeopardize the success of the implant. The reduced prognosis of these patients, the greatest risk of infections and the high cost of the procedure are the reasons for the scarce use of this technique in this patient setting. Unfortunately, changes of the immune system occurring in patients with lymphoproliferative diseases may favor the onset of intractable persistent chronic pain. Furthermore, some treatments like radiotherapy, chemotherapy, and the administration of monoclonal antibodies may further impair the immune defense of these patients and generate chronic inflammatory phenomena acting as a substrate for the development of persistent chronic pain. Some surgical diagnostic techniques like VATS in our patient 1 may contribute to generate a chronic persistent pain syndrome. Therefore, in patients with lymphoproliferative diseases, there are reasons inducing a persistent chronic pain not responsive to usual treatments for whom the SCS should be taken into consideration.

All five patients in this study were in good general condition, with a PS of 2, and had an indolent lymphoproliferative disease, and for all of them, a survival of 5 years or more was expected. The implant and use of the SCS in a one-shot procedure were safe in all five treated patients who did not develop adverse events and infections. Of note, this procedure is reversible in case of ineffectiveness (43).

The habit to deprive patients with lymphoproliferative diseases of the possibility of being treated with SCS just because for them a reduced survival duration is expected is extremely reductive and frustrating. Even if in an evaluation of the cost-effectiveness ratio, the expected duration of life has a relevant role, it is inhumane not to reserve also for patients with indolent lymphoproliferative diseases the possibility to take advantage of the benefits SCS may offer. All this considering, there is no reason to deny these patients the possibility of accessing SCS treatment. The data presented in this study might confirm this opinion, but further studies are needed to conclude on this topic.

## Data Availability Statement

The raw data supporting the conclusions of this article will be made available by the authors, without undue reservation.

## Ethics Statement

Written, informed consent was obtained for the publication of these case reports.

## Author Contributions

AS and AP: conceptualization. AS, CS, MD, PB, AMS, ESag, ESar, and DT: data curation and investigation. AS and BC: methodology. AS: project administration, writing—original draft, and writing—review and editing. All authors have read and agreed to the published version of the manuscript.

## Conflict of Interest

The authors declare that the research was conducted in the absence of any commercial or financial relationships that could be construed as a potential conflict of interest.

## Abbreviations

SCS: subcutaneous spinal cord stimulation
HFSCS: high-frequency SCS without paraesthesia
FBSS: failed back surgery syndrome
CLL/SLL: chronic lymphocytic leukemia/small lymphocytic lymphoma
WM: Waldenström macroglobulinemia
FL: follicular lymphoma
MZL: marginal zone lymphoma
IGHV: immunoglobulin heavy chain region variable
LDH: lactate dehydrogenase
CLL-IPI: The International Prognostic Score
ISSWM: International Prognostic Scoring System
MPQ: McGill Pain Questionnaire
NRS: Numerical Rating Scale
PS: performance status
DRG: dorsal root ganglion
PRF: pulsed radiofrequency treatment.

## References

[1] de Miguel M Kraychete DC Meyer Nascimento RJ. Chronic pain: cytokines, lymphocytes and chemokines. Inflamm Allergy Drug Targets. (2014) 13:339–49. 10.2174/1871528114666150114170004 25587846

[2] Scholz J Woolf CJ The neuropathic pain triad: neurons, immune cells and glia. Nature Neurosci. (2007) 10:1361–68. 10.1038/nn1992 17965656

[3] Zhang JM An J Cytokines, inflammation, and pain. Int Anesthesiol Clin. (2007) 45:27–37. 10.1097/AIA.0b013e318034194e 17426506 PMC2785020

[4] Sica A Casale B Spada A Di Dato MT Sagnelli C Calogero A Differential diagnosis: retroperitoneal fibrosis and oncological diseases. Open Med. (2019) 15:22–6. 10.1515/med-2020-0005 PMC6944454 31922016

[5] Basbaum AI Bautista DM Scherrer G Julius D. Cellular and molecular mechanisms of pain. Cell. (2009) 139:267–84. 10.1016/j.cell.2009.09.028 19837031 PMC2852643

[6] Uçeyler N Rogausch JP Toyka KV Sommer C. Differential expression of cytokines in painful and painless neuropathies. Neurology. (2007) 69:42–9. 10.1212/01.wnl.0000265062.92340.a5 17606879

[7] Sica A Vitiello P Papa A Calogero A Sagnelli C Casale D. Use of rituximab in NHL malt type pregnant in I° trimester for two times. Open Med. (2019) 14:757–60. 10.1515/med-2019-0087 31844674 PMC6884922

[8] Uçeyler N Eberle T Rolke R Birklein F Sommer C. Differential expression patterns of cytokines in complex regional pain syndrome. Pain. (2007) 132:195–205. 10.1016/j.pain.2007.07.031 17890011

[9] Koch A Zacharowski K Boehm O Stevens M Lipfert P von Giesen HJ. Nitric oxide and pro-inflammatory cytokines correlate with pain intensity in chronic pain patients. Inflamm Res. (2007) 56:32–7. 10.1007/s00011-007-6088-4 17334668

[10] Sica A Casale B Di Dato MT Calogero A Spada A Sagnelli C Cancer and not cancer related chronic pain: from the physiopathological bases to the management. Open Med. (2019) 14:761–66. 10.1515/med-2019-0088 PMC6795027 31637307

[11] Capace M Creta M Calogero A La Rocca R Napolitano L Barone B. Does physical activity regulate prostate carcinogenesis and prostate cancer outcomes? A narrative review. Int J Environ Res Public Health. (2020) 17:E1441. 10.3390/ijerph17041441 32102283 PMC7068391

[12] Sandkühler J Gruber-Schoffnegger D. Hyperalgesia by synaptic long-term potentiation (LTP): an update. Curr Opin Pharmacol. (2012) 12:18–27. 10.1016/j.coph.2011.10.018 22078436 PMC3315008

[13] Gruber-Schoffnegger D Drdla-Schutting R Hönigsperger C Wunderbaldinger G Gassner M Sandkühler J. Induction of thermal hyperalgesia and synaptic long-term potentiation in the spinal cord lamina I by TNF-α and IL-1β is mediated by glial cells. J Neurosci. (2013) 33:6540–51. 10.1523/JNEUROSCI.5087-12.2013 23575851 PMC6619063

[14] Caccavale S Vitiello P Franco R Panarese I Ronchi A Sica A. Dermoscopic characterization of folliculotropic mycosis fungoides selectively localized on trunk and limbs. Int J Dermatol. (2019) 58:e187–9. 10.1111/ijd.14490 31135956

[15] Thacker M. A Clark A.K Marchand F McMahon S.B. Pathophysiology of peripheral neuropathic pain: immune cells and molecules. Anesth Analg. (2007) 105:838–47. 10.1213/01.ane.0000275190.42912.37 17717248

[16] Merskey H Bogduk N Classification of Chronic Pain. 2nd ed Chair; Seattle: IASP Press (1994). p. 1.

[17] Vellucci R Heterogeneity of chronic pain. Clin Drug Investig. (2012) 32(Suppl. 1):3–10. 10.2165/11630030-000000000-00000 22356219

[18] Calogero A Sagnelli C Carlomagno N Tammaro V Candida M Vernillo A. Familial polyposis coli: the management of desmoid tumor bleeding. Open Med. (2019) 14:572–6. 10.1515/med-2019-0064 31410368 PMC6689203

[19] Cascone R Sica A Sagnelli C Carlucci A Calogero A Santini M. Endoscopic treatment and pulmonary rehabilitation for management of lung abscess in elderly lymphoma patients. Int J Environ Res Public Health. (2020) 17:E997. 10.3390/ijerph17030997 32033391 PMC7038113

[20] Fiorelli A D'Andrilli A Carlucci A Vicidomini G Loizzi D Ardò NP. Prognostic factors of lung cancer in lymphoma survivors (the LuCiLyS study). Transl Lung Cancer Res. (2020) 9:90–102. 10.21037/tlcr.2019.12.28 32206557 PMC7082280

[21] Bell GK Kidd D North RB. Cost-effectiveness analysis of spinal cord stimulation in treatment of failed back surgery syndrome. J Pain Symptom Manage. (1997) 13:286–95. 10.1016/S0885-3924(96)00323-5 9185434

[22] Floridia D Cerra F Guzzo G Marino S Muscarà N Corallo F. Treatment of pain post-brachial plexus injury using high-frequency spinal cord stimulation. J Pain Res. (2018) 11:2997–3002. 10.2147/JPR.S168031 30568480 PMC6267358

[23] Reginelli A Belfiore MP Russo A Turriziani F Moscarella E Troiani T. A preliminary study for quantitative assessment with HFUS (High Frequency ultrasound) of nodular skin melanoma Breslow thickness in adults before surgery: interdisciplinary team experience. Curr Radiopharm. (2020) 13:48–55. 10.2174/1874471012666191007121626 31589132

[24] Jeon YH. Spinal cord stimulation in pain management: a review. Korean J Pain. (2012) 25:143–50. 10.3344/kjp.2012.25.3.143 22787543 PMC3389317

[25] Viscardi G Zanaletti N Ferrara MG Sica A Falcone U Guastafierro S. Atypical haemolytic-uraemic syndrome in patient with metastatic colorectal cancer treated with fluorouracil and oxaliplatin: a case report and a review of literature. ESMO Open. (2019) 4:e000551. 10.1136/esmoopen-2019-000551 31673427 PMC6802959

[26] Aljuboori Z Meyer K Sharma M Ball T Nauta H. Cost comparison among punctate midline myelotomy, intrathecal pain pump, and spinal cord epidural stimulator. Surg Neurol Int. (2020) 11:25. 10.25259/SNI_16_2020 32123613 PMC7049879

[27] Ghosh P Gungor S. Utilization of concurrent dorsal root ganglion stimulation and dorsal column spinal cord stimulation in complex regional pain syndrome. Neuromodulation. (2020) 10.1111/ner.13144. [Epub ahead of print]. 32162402

[28] Sica A Vitiello P Caccavale S Sagnelli C Calogero A Doraro CA. Primary cutaneous DLBCL non-GC type: challenges of a rare case. Open Med. (2020) 15:119–125. 10.1515/med-2020-0018 32258414 PMC7101477

[29] Kumar K Bishop S. Financial impact of spinal cord stimulation on the healthcare budget: a comparative analysis of costs in Canada and the United States. J Neurosurg Spine. (2009) 10:564–73. 10.3171/2009.2.SPINE0865 19558289

[30] Sica A Vitiello P Sorriento A Ronchi A Calogero A Sagnelli C Lymphomatoid papulosis: overiew. Minerva Med. (2020) 111:166–72. 10.23736/S0026-4806.19.06395-X 31958921

[31] Sica A Vitiello P Ronchi A Casale B Calogero A Sagnelli E. Primary cutaneous anaplastic large cell lymphoma (pcALCL) in elderly, the importance of a sport activity training. Int J Environ Res Public Health. (2020) 17:E839. 10.3390/ijerph17030839 32013101 PMC7037068

[32] Ronchi A Zito Marino F Vitiello P Caccavale S Argenziano G Crisci S A case of primary cutaneous B-cell lymphoma with immature features in an old man. Diffuse Large B-cell Lymhpoma with immature features or B-cell Lymhpoblastic Lymphoma? J Cutan Pathol. (2020) 10.1111/cup.13795. [Epub ahead of print]. 32623764

[33] Sica A Sagnelli C Papa A Ciccozzi M Calogero A Martinelli E An anecdotal case report of chronic lymphatic leukemia with del(11q) treated with ibrutinib: artificial nourishment and physical activity program. Int J Environ Res Public Health. (2020) 17 10.3390/ijerph17061929 PMC7142487 32188040

[34] Vitiello P Sica A Ronchi A Caccavale S Franco R Argenziano G. Primary cutaneous B cell lymphomas: an update. (2020) Front Oncol. 10:651. 10.3389/fonc.2020.00651 32528871 PMC7266949

[35] North R Desai MJ vangeneugden J Raftopoulos C van Havenbergh T Deruytter M- Postoperative infections associated with prolonged spinal cord stimulation trial duration (PROMISE RCT). Neuromodulation. (2020) 23:620–5. 10.1111/ner.13141 32267989 PMC7496399

[36] Merli M Frigeni M Alric L Visco C Besson C Mannelli L. Direct-acting antivirals in hepatitis c virus-associated diffuse large B-cell lymphomas. Oncologist. (2018) 24:e720–9. 10.1634/theoncologist.2018-0331 30552159 PMC6693710

[37] Coppola N Pisaturo M Guastafierro S Tonziello G Sica A Iodice V. Increased hepatitis C viral load and reactivation of liver disease in HCV RNA-positive patients with onco-haematological disease undergoing chemotherapy. Dig Liver Dis. (2012) 44:49–54. 10.1016/j.dld.2011.07.016 21885355

[38] Pisaturo M Guastafierro S Filippini P Tonziello G Sica A Di Martino F. Absence of occult HCV infection in patients experiencing an immunodepression condition. Infez Med. (2013) 21:296–301. 24335460

[39] Coppola N Pisaturo M Guastafierro S Tonziello G Sica A Sagnelli C. Absence of occult hepatitis C virus infection in patients under immunosupressive therapy for oncohematological diseases. Hepatology. (2011) 54:1487–9. 10.1002/hep.24436 21608002

[40] Bagaglio S Uberti-Foppa C Sagnelli C Lai A Hasson H Salpietro S. HIV-1 recombinant forms in immigrants regularly residing in Milan, northern Italy. Infection. (2020) 48:553–8. 10.1007/s15010-020-01434-3 32430647

[41] Merli M defrancesco I Visco C Besson C Di Rocco A Arcari A Direct-acting antivirals in relapsed or refractory hepatitis C virus-associated diffuse large B-cell lymphoma. Leuk Lymphoma. (2020) 61:2122–8. 10.1080/10428194.2020.1755859 32343165

[42] Sica A Casale D Rossi G Casale B Ciccozzi M Fasano M. The impact of the SARS-CoV-2 infection, with special reference to the haematological setting. J Med Virol. (2020). 10.1002/jmv.26197. [Epub ahead of print]. 32558961 PMC7323149

[43] Ma BB Rao VR. Responsive neurostimulation: candidates and considerations. Epilepsy Behav. (2018) 88:388–95. 10.1016/j.yebeh.2018.09.032 30355456

